# Comparison of Chemical Composition and Biological Activities of Eight *Selaginella* Species

**DOI:** 10.3390/ph14010016

**Published:** 2020-12-26

**Authors:** Bára Křížkovská, Rohitesh Kumar, Kateřina Řehořová, David Sýkora, Simona Dobiasová, Denisa Kučerová, Maria Carmen Tan, Virgilio Linis, Glenn Oyong, Tomáš Ruml, Jan Lipov, Jitka Viktorová

**Affiliations:** 1 Department of Biochemistry and Microbiology, University of Chemistry and Technology Prague, Technická 5, 166 28 Prague, Czech Republic; krizkova@vscht.cz (B.K.); rohiteshkumar@gmail.com (R.K.); rehorova@vscht.cz (K.Ř.); dobiasos@vscht.cz (S.D.); kuceroad@vscht.cz (D.K.); rumlt@vscht.cz (T.R.); lipovj@vscht.cz (J.L.); 2 Department of Analytical Chemistry, University of Chemistry and Technology Prague, Technická 5, 166 28 Prague, Czech Republic; sykorad@vscht.cz; 3 Department of Chemistry, De La Salle University, 2401 Taft Avenue, Manila 1004, Philippines; maria.carmen.tan@dlsu.edu.ph; 4 Department of Biology, De La Salle University, 2401 Taft Avenue, Manila 1004, Philippines; virgiliolinis@gmail.com; 5 Molecular Science Unit Laboratory, Center for Natural Sciences and Environmental Research, De La Salle University, 2401 Taft Avenue, Manila 1004, Philippines; glenn.oyong@dlsu.edu.ph

**Keywords:** *Selaginella*, selaginellins, flavonoids, cytotoxicity, AChE inhibition, anti-inflammatory

## Abstract

*Selaginella* P. Beauv. is a group of vascular plants in the family Selaginellaceae Willk., found worldwide and numbering more than 700 species, with some used as foods and medicines. The aim of this paper was to compare methanolic (MeOH) and dichloromethane (DCM) extracts of eight *Selaginella* species on the basis of their composition and biological activities. Six of these *Selaginella* species are underinvestigated. Using ultra-high performance liquid chromatography–high-resolution mass spectrometry (UHPLC–HRMS) analysis, we identified a total of 193 compounds among the tested *Selaginella* species, with flavonoids predominating. MeOH extracts recovered more constituents that were detected, including selaginellins, the occurrence of which is only typical for this plant genus. Of all the tested species, *Selaginella* *apoda* contained the highest number of identified selaginellins. The majority of the compounds were identified in *S. apoda*, the fewest compounds in *Selaginella* *cupressina*. All the tested species demonstrated antioxidant activity using oxygen radical absorption capacity (ORAC) assay, which showed that MeOH extracts had higher antioxidant capacity, with the half maximal effective concentration (EC_50_) ranging from 12 ± 1 (*Selaginella* *myosuroides*) to 124 ± 2 (*Selaginella* *cupressina*) mg/L. The antioxidant capacity was presumed to be correlated with the content of flavonoids, (neo)lignans, and selaginellins. Inhibition of acetylcholinesterase (AChE) was mostly discerned in DCM extracts and was only exhibited in *S. myosuroides*, *S. cupressina*, *Selaginella* *biformis*, and *S. apoda* extracts with the half maximal inhibitory concentration (IC_50_) in the range of 19 ± 3 to 62 ± 1 mg/L. Substantial cytotoxicity against cancer cell lines was demonstrated by the MeOH extract of *S. apoda*, where the ratio of the IC_50_ HEK (human embryonic kidney) to IC_50_ HepG2 (hepatocellular carcinoma) was 7.9 ± 0.2. MeOH extracts inhibited the production of nitrate oxide and cytokines in a dose-dependent manner. Notably, *S. biformis* halved the production of NO, tumor necrosis factor (TNF)-α, and interleukin (IL)-6 at the following concentrations: 105 ± 9, 11 ± 1, and 10 ± 1 mg/L, respectively. Our data confirmed that extracts from *Selaginella* species exhibited cytotoxicity against cancer cell lines and AChE inhibition. The activity observed in *S. apoda* was the most promising and is worth further exploration.

## 1. Introduction

Selaginellaceae Willk. (spikemoss), Lycopodiaceae P. Beauv. ex Mirb. (clubmosses), and Isoetaceae Dumort. (quillworts) collectively fall under the division Lycopodiophyta D.H.Scott, which is the oldest lineage of vascular plants surviving on earth [1,2]. *Selaginella* P. Beauv., which is the only surviving genus in the family Selaginellaceae, has more than 700 known species, which are mostly found in tropical and subtropical regions around the globe [1,3]. *Selaginella* species are mainly adapted to these xeric regions by their surface composition (the presence of waxes [4]) and by the ability of shoots to roll themselves up during a drought [4,5]. The genus *Selaginella* is divided into seven subgenera [6]. One of the most famous is *Selaginella lepidophylla* (Hook. & Grev.) Spring (commonly called the false rose of Jericho), a poikilohydric plant that has the ability to survive extreme dehydration and resurrect after a long period without water. This ability is supported by a huge concentration of trehalose, which helps to protect some of the proteins and membranes during a drought [7]. Another important example is *Selaginella apoda* (L.) C. Morren [8], which has all the prerequisites for being a model organism of lycophytes, mainly because of its short life cycle.

Because of their pharmacological activities, several species of *Selaginella* have been widely used among many cultures across the world as herbal and traditional medicines [1] for the treatment of inflammation [9], dysmenorrhea [10], chronic hepatitis [9], and hyperglycemia [2]. These activities were demonstrated mainly in *Selaginella tamariscina* (P. Beauv.) Spring, *Selaginella moellendorffii* Hieron, *Selaginella pulvinata* (Hook. & Grev.) Maxim., and *Selaginella doederleinii* Hieron, which are among the most studied plants from this genus. The essential oils of *Selaginella delicatula* (Desv. ex Poir.) Alston were shown to exhibit antimicrobial activity, especially against *Escherichia coli*, *Pseudomonas aeruginosa*, and *Corynebacterium pyogenes* [11]. Neuroprotective activity was also detected in in vivo testing of the aqueous extract of this species [12,13]. Biflavonoids isolated from *S. delicatula* also exhibited significant cytotoxicity against various tumor cell lines [14]. *Selaginella uncinata* is well known due to the blue color of the leaves caused by an anthocyanin named delphinidin [15]. An extract of this plant showed antiproliferative activity on human liver cells (Bel-7402) and human cervical carcinoma cells (HeLa) [16]. In addition, ethanolic extract of *S. uncinata* (Desv.) Spring demonstrated promising anti-anoxic activity [17]. Flavonoids from *S. uncinata* (Desv.) Spring showed the inhibition of airway inflammation in vivo [18].

**Figure 1:**
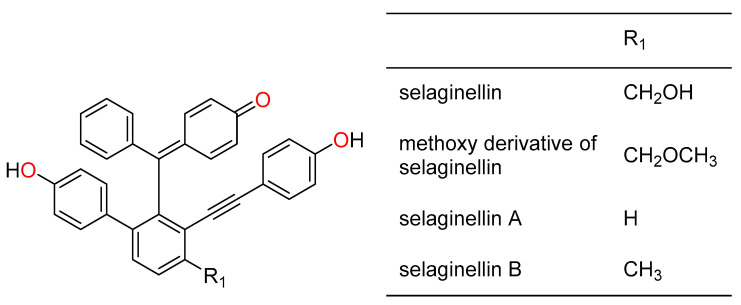
Chemical structures of selaginellin, methoxy derivative of selaginellin, and selaginellins A–B.

Chemical investigation of *Selaginella* has resulted in the identification of various new compounds such as flavonoids [19,20], lignans [21,22], alkaloids [23], and terpenoids [24], which have exhibited a wide range of biological activities, including antioxidant [25,26], antidiabetic [2], anticancer [24,27], and antimicrobial [28,29] effects. A group of unique compounds named selaginellins, which possess a *p*-quinone methide and alkynylphenol functional groups, have also been identified from various species of *Selaginella* [30]. Selaginellins are predominantly colored compounds; the first selaginellin (Figure 1) was isolated as a racemic mixture from *Selaginella sinensis* [31]. Due to its structural complexity, selaginellin crystallization was unsuccessful until a crystalline methoxy derivative of selaginellin was synthesized and the structure was determined using X-ray crystallographic data analysis [31]. Shortly after this discovery, the second and third members of this interesting class of compounds, selaginellins A (**2**) and B (**3**), were isolated from *S. tamariscina* [32]. These compounds have antioxidant potential and cytotoxic activity against human cervical carcinoma cells [33]. To date, more than 60 selaginellins and selaginpulvilin analogues have been reported [34], and have been shown to exhibit various biological activities such as cytotoxicity [33,35], phosphodiesterase-4 (PDE4) inhibitory activity [36], protein tyrosine phosphatase 1B (PTP1B) inhibitory activity [37], antimicrobial activity [38], and antidiabetic activity [2]. Selaginellin derivatives exhibited antimicrobial activity against *Staphylococcus aureus* and antifungal activity against *Candida albicans* [29]. Here, we report the chemical composition and biological activities of eight underinvestigated *Selaginella* species.

## 2. Results

### 2.1. Chemical Composition

By using the ultra-high performance liquid chromatography–high-resolution mass spectrometry (UHPLC–HRMS) analysis of dichloromethane and methanol crude extracts of *Selaginella*, we identified 193 known secondary metabolites (Table 1 and Table 2) and found an additional 30 unidentified compounds. Approximately 60% of the unidentified compounds were present in the dichloromethane (DCM) extracts (see Table S1 for full list of compounds). In total, eight different classes of compounds were present in the dichloromethane extracts, while nine classes were found in the methanolic extracts. Due to their polar nature, no alkaloids were detected in the dichloromethane extracts. Of the known compounds in the methanolic extract of *Selaginella* (Table 1), flavonoids were found to be the major constituents, followed by lignans, phenolics, and selaginellins in that order. Moreover, the methanolic extract of *S. apoda* possessed the highest number of secondary metabolites amongst the species of *Selaginella* analyzed in this study. A total of 25 different selaginellins and selaginellin derivatives were also identified in the methanolic and dichloromethane extracts.

### 2.2. Antioxidant Activity

**Figure 2:**
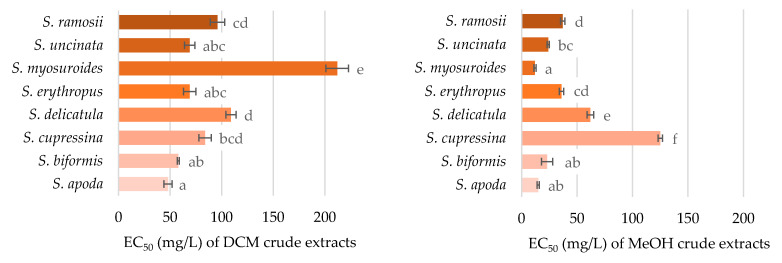
Antioxidant activity, measured by oxygen radical absorption capacity (ORAC) assay, is presented as concentrations (mg/L) of crude extracts that halved the amounts of free oxygen radicals (EC_50_). Data are presented as the average of three repetitions ± standard error of the mean. Letters indicate the differences between the groups (ANOVA followed by Tukey’s post hoc test, *p* < 0.05) within one assay. Statistically significant levels are denoted with different letters.

The antioxidant activity of the crude extracts was investigated to observe their scavenging potential of oxygen radicals. As can be seen in Figure 2, all the tested extracts had some degree of radical quenching activity. Comparisons of the extraction solvent techniques showed that methanolic extracts were stronger antioxidants than dichloromethane extracts (confirmed by one-way ANOVA with Tukey’s post hoc test with *p* < 0.01). This is congruent with the chemical analyses, which detected a higher concentration of flavonoids, known for their antioxidant activity, in the methanolic extracts. More pronounced antioxidant capacity was seen in *Selaginella myosuroides*, *Selaginella biformis*, and *S. apoda* methanolic extracts. Unlike *S. myosuroides*, *Selaginella cupressina* methanolic extract was almost 10 times less active, and was the least potent species for radical quenching. The antioxidant function of each species was in accordance with the content of flavonoids, (neo)lignans, and selaginellins, and was further substantiated by the values of Pearson correlation coefficient being equal to 0.513, 0.573, and 0.522, respectively, while the critical value was 0.497 (*n* = 16, df = 14, α = 0.05). However, no correlation was obtained between the antioxidant activity and the detected compound.

### 2.3. Cytotoxicity against Cancer Cell Lines

**Figure 3:**
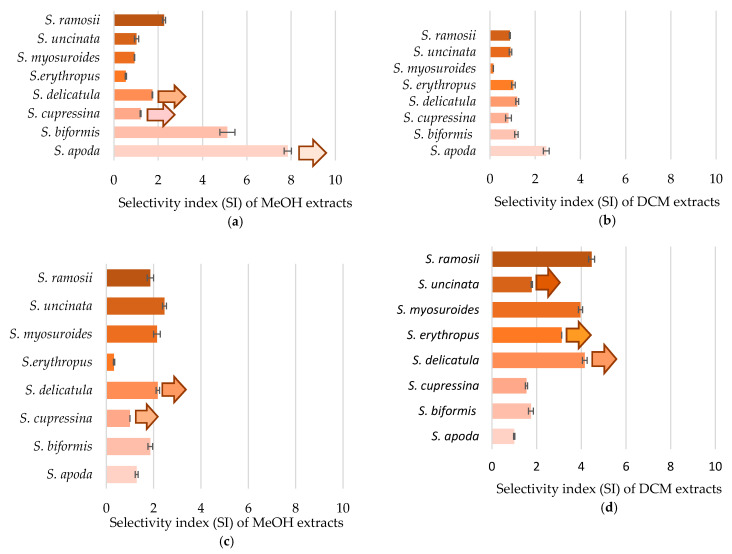
Cytotoxicity against cancer cell lines of the crude extracts expressed as the selectivity index: (**a**) ratio of IC_50_ values of HEK293T and HepG2 cell lines; (**b**) ratio between IC_50_ for HEK293T and HepG2 cell lines; (**c**) ratio between IC_50_ for HaCat and HeLa cell lines; (**d**) ratio between IC_50_ for HaCat and HeLa cell lines. Data are presented as the average of three repetitions ± standard error of the mean. The arrows indicate the minimum values of the selective indexes, where the IC_50_ value for the control line was not reached even at the highest tested concentration (500 mg/L).

Cytotoxicity against cancer cell lines was determined as the selectivity index, which represents the differences between the cytotoxicity for normal (HEK293T, HaCat) cells and for cancer (HepG2, HeLa) cells (Figure 3). The selectivity index (SI) was calculated as the ratio of the extract’s concentration that halved the viability of the control cell line to the concentration that halved the viability of the cancer cell line. The higher the SI, the higher the selectivity of the extract to affect the viability of cancerous cells and not adversely affect normal cells. The SI was calculated for the cell lines derived from internal organs (kidney and liver) and for epithelial tissues (dermal fibroblasts and cervix). All the tested extracts possess cytotoxicity against cancer cell lines. Interestingly, methanolic extracts were more selective for the organ-derived carcinoma, while dichloromethane extracts were more selective against epithelial carcinoma. The highest selectivity index was determined for the methanolic extract of *S. apoda* (SI > 7.9 for HEK293T and HepG2), followed by *S. biformis* (SI = 5.1) and the dichloromethane extracts of *Selaginella ramosii* (SI = 4.5) and *S. delicatula* (SI > 4.2).

Both the selectivity index and IC_50_ values corresponding to each cell line were associated with the 193 compounds identified by chemical analyses of the respective methanolic and dichloromethane extracts. When plotting the results for all 16 extracts tested, we also determined the Pearson’s correlation coefficient. Table 3 shows the chemical compounds which correlated with the cytotoxicity against cancer cell lines with a significance level (α) either equal or lower than 0.05. The cytotoxicity of crude extracts against human cervical adenocarcinoma correlated with five compounds identified in the methanolic extract, four of them belonging to selaginellins and their derivatives. Cytotoxicity against cancer cell lines also correlated with seven flavonoids, three phenols, two lignans, and one representative of each of the following metabolites: terpenoid, fatty acid, quinone, and steroid. The amount of chalcone correlated with cytotoxicity against cancer cell lines with the highest significance (α < 0.01).

Pearson’s correlation coefficient was determined by correlating the cytotoxicity against cancer cell lines and total amount of secondary metabolite classes. The dependence of the selectivity index obtained from organ-derived cell lines and either selaginellins or quinones showed a significant correlation, with coefficients equal to 0.537 (*n* = 16, df = 14, crit. value = 0.497, α = 0.05) and 0.632 (*n* = 15, df = 13, crit. value = 0.592, α = 0.02), respectively.

### 2.4. Inhibition of Acetylcholinesterase Activity

**Figure 4:**
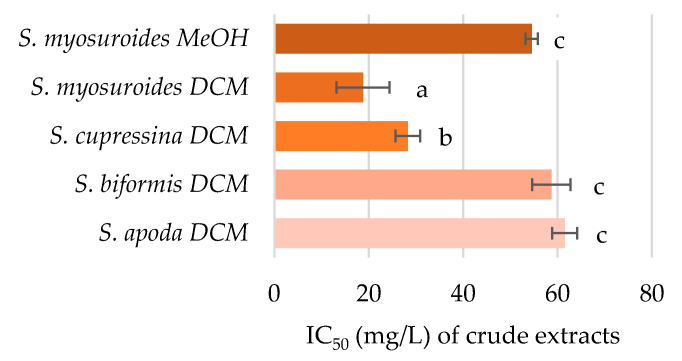
Inhibitory effect of *Selaginella* extracts on acetylcholinesterase activity expressed as IC_50_ values. Data are presented as the average of four repetitions ± standard error of the mean. Letters indicate the differences between the groups (ANOVA followed by Tukey’s post hoc test, *p* < 0.05) within one assay. Statistically significant levels were denoted with different letters.

In contrast to previously shown results, the inhibition of acetylcholinesterase was demonstrated mainly by dichloromethane extracts (Figure 4). The only methanolic extract capable of inhibition of this cholinergic enzyme was the extract of *S. myosuroides*; similarly, its dichloromethane extract was the most active among the samples. The DCM extract of this species was almost 1.5 times more active than that of *S. cupressina*, and three times more active than *S. biformis* and *S. apoda* DCM extracts. The remainder of the extracts exhibited no activity, even at the highest stipulated concentration (62.5 mg/L). The inhibition of acetylcholinesterase can be attributed to selaginellins on the basis of the Pearson’s correlation (value = 0.952, *n* = 4, df = 2, crit. value = 0.950, α = 0.05).

### 2.5. Anti-Inflammatory Activity

**Figure 5:**
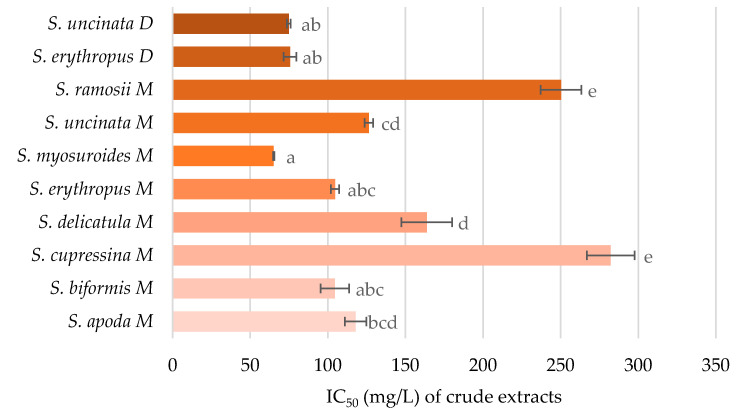
Inhibition of nitrate oxide production. Data are presented as the average of three repetitions ± standard error of the mean. Letters indicate the differences between the groups (ANOVA followed by Tukey’s post hoc test, *p* < 0.05) within one assay. Statistically significant levels were denoted with different letters.

The anti-inflammatory activity of crude extracts was tested as the ability to reduce nitrate oxide production and the release of cytokines. Activity affecting nitrate oxide production was observed primarily in methanolic extracts of *S. myosuroides*, *Selaginella erythropus*, and *S. biformis* (Figure 5). Only DCM extracts of *S. uncinata* and *S. erythropus* exhibited any activity. The inhibition of nitrate oxide production was related to the total (neo)lignans content, specifically seladoeflavone E (flavonoid) and selamoellenin A (lignin). The correlation coefficients are presented in Table 4.

**Figure 6:**
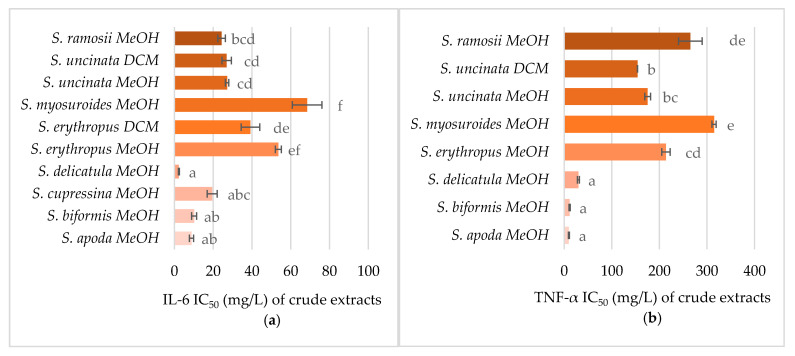
The effect of *Selaginella* extracts on (**a**) interleukin (IL)-6 and (**b**) tumor necrosis factor (TNF)-α levels in RAW 264.7 cells. Data are presented as the average of three repetitions ± standard error of the mean. Letters indicate the differences between the groups (ANOVA followed by Tukey’s post hoc test, *p* < 0.05) within one assay. Statistically significant levels were denoted with different letters.

As several *Selaginella* species exhibited a promising reduction of nitrate oxide production, their ability to inhibit the release of cytokines was also tested to further validate the results. As can be seen in Figure 6, all methanolic extracts inhibited tumor necrosis factor (TNF)-α and interleukin (IL)-6 production in a dose-dependent manner; however, the production of cytokines was only modulated by two DCM extracts—*S. uncinata* and *S. erythropus*. The IL-6 production was more sensitive to the extracts than that of TNF-α. The most potent species for inhibiting cytokine release were *S. apoda*, *S. biformis*, *S. delicatula*, and *S. cupressina*. The correlations showed that the upregulation of IL-6 was affected by *Selaginella*-unique compounds: selagin (flavonoid), pulvinataphendiol (selaginellin), and further by daucosterol—a natural phytosterol-like compound and derivative of phenylpropanoic acid. Selagin, pulvinataphendiol, and the natural phytosterol-like compound also inhibited the expression of TNFα resembling the *Selaginella*-unique biflavonoid isomers (robustaflavone 4′-methyl ether, podocarpusflavone, neocryptomerin, sequoiaflavone, isocryptomerin, or sotetsuflavone).

## 3. Discussion

Plant bioprospecting is a process leading to the exploration of natural plant species for biologically active compounds, which could be further isolated or chemically synthetized and used in any sector of the market economy for profit. Although many of these compounds have found applications in the pharmaceutical industry, in many cases the bioprospecting of medical plants has failed. Usually, the reason for the failure lies in the fact that the isolation of pure compounds usually negates the additive or synergistic effect of the additive compounds present in the extract. Therefore, over the last decade, attention has been refocused back on the use of more complex crude or partially purified extracts.

Of all the *Selaginella* species, *S. tamariscina* (to date, 144 publications in Web of Science) is the one that is best characterized for its biological activities and composition. Herein, we focused on some lesser-known representatives of *Selaginella* species, including *S. uncinata* (31 publications), which was reported in more than half of the total publications, followed by *S. delicatula* (12 publications), *S. apoda* (8 publications), *S. erythropus* (7 publications), *S. biformis* (1 publication), and species that have not been reported before, namely, *S. cupressina*, *S. myosuroides*, and *S. ramosii*.

It is well documented in the literature that the genus *Selaginella* produces a wide range of structurally different secondary metabolites such as flavonoids [19,20] and lignans [21,22], together with their unique pigments, selaginellins [27,29,30,32,36]. Our MS data analysis (see Table S1) also confirmed that flavonoids, (neo)lignans, and selaginellins are among the most common natural products identified in both the methanolic and dichloromethane extracts of *Selaginella.* The majority of the identified flavonoids (90%), (neo)lignans (81%), and selaginellins (79%) were present in the methanolic extracts compared to flavonoids (10%), (neo)lignans (19%), and selaginellins (21%) in the dichloromethane extracts, respectively. Selaginellin; selaginellins D, F, O, P, Q, S, and W; selaginpulvilins A, B, E, F, I, K, L, N, O, and T; with selariscins A, and B; and selariscinins B, and D were the unique compounds identified from both the DCM and methanolic (MeOH) extracts. Selaginellin was initially isolated from *S. sinensis* [31], selaginellin S from *S. moellendorffii* Hieron [40]; selaginellin O [33], selaginpulvilin N [36], selaginpulvilin O [36], selaginpulvilin T [36], selariscin A [36], selariscin B [36], selariscinin B [3], and selariscinin D [3] from *S. tamariscina*; and selaginellin D [29], selaginellin F [29], selaginellin P [30], selaginellin Q [30], selaginpulvilin A [41], selaginpulvilin B [41], selaginpulvilin E [42,43], selaginpulvilin F [43], selaginpulvilin I [43], selaginpulvilin K [44], and selaginpulvilin L [44] were isolated for the first time from *S. pulvinata.*

Several compounds identified here have not been previously reported for any of the *Selaginella* plants used in this study. Interestingly, only 12 unidentified compounds were detected in the methanolic extract, while 18 unidentified compounds were observed in the dichloromethane extract, indicating a potential plethora of new bioactive compounds from the *Selaginella* samples tested in this study.

The biological activity of tested samples is usually compared with standard compound(s) that have been reported to either exhibit the activity being assessed or used as a quantitative tool for comparison on the test samples. However, some researchers consider such distinctions to be inappropriate for crude extracts, as many compounds without activity usually dilute the effect or interfere with biologically active compounds. In contrast to the above statements, the combinatory action observed in complex matrices such as synergism/antagonism has been observed to affect the results. In addition, the concentrations of standards are usually prepared in a molar concentration, while the crude extracts can only be presented in weight (either wet biomass or dry weight) concentration. On the other hand, calibration curves or standards are usually shown to be a good laboratory praxis representing a validated methodology. Therefore, the standards for our methods are presented in Figure S1.

All of the tested *Selaginella* species demonstrated antioxidant behavior, cytotoxicity against cancer cell lines, and anti-inflammatory activities. The antioxidant activity of *Selaginella* species was facilitated by flavonoids, (neo)lignans, and selaginellins. We evaluated the correlation with antioxidant activity and identified compounds as well as the correlation of antioxidant activity and amount of secondary metabolite classes. The first mentioned correlation showed no hit with a significance ≤ 0.05, which is in agreement with the additive/synergistic mode of action of compounds in such complex matrixes as plant crude extracts [45]. The antioxidant activity of flavonoids is well known for flavonoids isolated from *Selaginella* species [46], but the antioxidant activity of (neo)lignans [47] and selaginellins [33] has not been verified yet. However, it has to be mentioned that biochemical assays have their limitations—they omit such important parameters as compound availability, first-pass metabolism, ability to cross the membranes, etc. Therefore, their results should be considered for information only. However, due to their price and speed, they are the most suitable alternative for high-throughput screening and first pre-selection.

The anticancer activity of *Selaginella* species is often reported as the toxicity of extracts for cancer cell lines; however, such a conclusion should not be established without appropriate controls. Therefore, here we report on the cytotoxicity against cancer cell lines of *Selaginella* species as a selectivity index (SI), which compares the concentration that halves the viability of the control cell line to the concentration that halves the viability of the cancerous cell line. Although the selectivity index has been known for more than a decade, it is still not commonly used, which hinders the uniform interpretation of results. The SI value reflects selectivity, where a higher SI indicates more selective cytotoxicity. An SI value lower than 2 indicates either low selectivity [48] or general toxicity [49]. Values higher than 2 indicate selectivity. According to Poirier et al. [50], SI = 5.5 indicates a good selectivity. The strictest criterion was applied by Peña-Morán [51], who considered the compound to be selective if SI ≥ 10. We determined the highest SI in methanolic extracts of *S. apoda* (SI > 7.9 ± 0.2) and *S. biformis* (SI = 5.1 ± 0.3) when applied to organ-derived cell lines. In contrast to organ-derived cell lines, where the methanolic extracts were more active, dichloromethane extracts were more active against epithelial carcinoma with SI = 4.5 ± 0.1, > 4.2 ± 0.1, and = 4.0 ± 0.1 for *S. ramosii*, *S. delicatula*, and *S. myosuroides*, respectively. The association of cytotoxicity against cancer cell lines and chemical composition showed a clear relationship, since up to 20 compounds were found to be responsible for the cytotoxicity against cancer cell lines of *Selaginella* species. Only a few of them have been already reported for their cytotoxicity against cancer cell lines, these being hexahydroxybiflavone [52], selaginellin O [33] and P [30], methyl cinnamate [53], seladoeflavone E [20], sinensioside A [54], oleic acid [55,56], chalcone [57], and phellodensin F [58].

The ability of *Selaginella* extracts to inhibit acetylcholinesterase has been previously tested by several groups. The methanolic extract of *Selaginella moellendorffii* exhibited no modulation of acetylcholinesterase (AChE) activity [23], which is in agreement with our results demonstrating the activity mainly by compounds extracted by DCM. The inhibition of AChE activity by a polar extract of *Selaginella delicatula* has been previously reported at a concentration higher than 50 mg/L [13], which is in line with our results, where the most active methanol-extracted species—*S. myosuroides*—had an AChE IC_50_ equal to 54.5 ± 1.3 mg/L.

Modulation of the immune system by *Selaginella* extracts were previously detected in some species. The oral administration of *S. moellendorffii* extract reduced the production of nitrate oxide and anti-inflammatory cytokines in rats [59]. Our results demonstrated a significant anti-inflammatory activity of selagin, which has been also previously demonstrated in vivo [60]. Immunoregulatory activity of daucosterol was also found both in vitro and in vivo [61,62,63]. *Selaginella*-unique biflavonoids also exhibited anti-inflammatory activity by reversing the TNF-α signaling pathway [64].

## 4. Materials and Methods

### 4.1. Material and Reagents

2,2′-Azo-bis-(2-methylpropionamidine) dihydrochloride (AAPH, Sigma-Aldrich, St. Louis, MO, USA); 2′,7′-dichlorofluorescin diacetate (DCFH-DA, Sigma-Aldrich); acetylcholinesterase (AChE, Sigma-Aldrich); 100× antibiotic antimycotic solution (Sigma-Aldrich); dichloromethane (Penta, Prague, Czech Republic); dimethylsulfoxide (DMSO, Sigma-Aldrich); Dulbecco’s modified Eagle’s medium–high glucose (DMEM, Sigma-Aldrich); Eagle’s minimum essential medium (EMEM, Sigma-Aldrich); Eagle’s minimal essential medium no phenol red (MEM, Sigma-Aldrich); fetal bovine serum (FBS, Sigma-Aldrich); fluorescein (Sigma-Aldrich); Griess reagent modified (Sigma-Aldrich); l-glutamine solution (Sigma-Aldrich); lipopolysaccharides from *Escherichia coli* O111:B4 (LPS, Sigma-Aldrich); methanol (Penta, Prague, Czech Republic); resazurin sodium salt (Sigma-Aldrich; trypsin–ethylenediaminetetraacetic acid (EDTA) solution (Sigma-Aldrich).

### 4.2. Plant Material

Eight *Selaginella* species were investigated in this study: *S. apoda* (L.) C. Morren, *S. biformis* A. Braun ex Kuhn, *S. cupressina* (Willd.) Spring, *S. delicatula* (Desv. ex Poir.) Alston, *S. erythropus* (Mart.) Spring, *S. myosuroides* (Kaulf.) Spring, *S. ramosii* Hieron, and *S. uncinata* (Desv. ex Poir.) Spring. The aerial parts of *S. apoda* (voucher specimen no. *Linis 5808–18*), an introduced Filipino *Selaginella* species, were gathered on 9 July 2018 from pots inside a plant nursery at Burnham Park, City of Baguio, province of Benguet. Meanwhile, the aerial parts of *S. biformis* (voucher specimen no. *Linis 5812–18*) were collected on 4 August 2018 from a moist, shaded soil bank beside a trail of a lowland forest on Mount Makiling, province of Laguna. For *S. cupressina* (voucher specimen no. *Linis 5811–18*), its aerial parts were collected on 14 July 2018 from a muddy soil bank of Pilis Creek inside a disturbed lowland forest of Mount Natib in the province of Bataan. Similarly, the aerial parts of *S. delicatula* (voucher specimen no. *Linis 5810–18*) were collected on the same date as *S. cupressina* (14 July 2018) from another muddy soil bank near Pilis Creek also inside a disturbed lowland forest of Mount Natib in the province of Bataan. *S. erythropus* (voucher specimen no. *Linis 5809–18*) is another introduced species in the Philippines. Its aerial parts were collected on 9 July 2018 from clay pots cultivated in a plant nursery in Burnham Park, Baguio City, Benguet Province, the Philippines. Aerial parts of *S. myosuroides* (voucher specimen no. *Linis 5813–18*), on the other hand, were collected on 4 August 2018 from a heavily shaded moist rockslide within a lowland forest of Mount Makiling in the province of Laguna, while aerial parts of *S. ramosii* (voucher specimen no. *Linis 5814–18*) were collected on 4 August 2018 from a steep eroded soil bank in the montane forest of the same mountain. Like *S. apoda* and *S. erythropus*, *S. uncinata* (voucher specimen no. *Linis 5807–18*) is the third introduced *Selaginella* species in the Philippines investigated in this study. Aerial parts of *S. uncinata* were collected on 5 June 2018 from a shady area of grassy, moist, humid ground inside De La Salle University, Manila. All eight *Selaginella* specimens were identified by Dr. V.C. Linis, a professor and botanist at De La Salle University. Voucher specimens were prepared, preserved, and deposited in De La Salle University Herbarium (code, DLSUH), with some duplicates sent to the Philippine National Herbarium (code, PNH) for further reference.

The collected *Selaginella* samples were all air-dried in an air-conditioned laboratory room (room temperature set to 17 °C). The samples were laid on wide, open cardboard boxes and left to air dry until visibly dry throughout.

### 4.3. Extraction of Secondary Metabolites

The dried and finely ground plant materials were extracted with DCM (2 × 100 mL) followed by MeOH extraction (2 × 100 mL) for 24 h, after which the solvents were removed under pressure to yield dried DCM and MeOH crude extracts. A solution of 1 g/L concentration for each crude extract (16 samples) was prepared, and the extracts were analyzed using UHPLC–HRMS. Samples were diluted 10 times (v/v) and injected into UHPLC using the starting mobile phase.

### 4.4. Determination and Identification of Secondary Metabolites

UHPLC–HRMS analysis was performed using an Accela Open autosampler (20 µL sample loop) and an Accela 600 pump (Thermo Fisher Scientific, Walham, MA, USA) coupled to the electrospray ion (ESI) source of an LTQ Orbitrap Velos mass spectrometer (Thermo Fisher Scientific, Walham, MA, USA) in positive and negative ion modes. HPLC separations were performed in a Phenomenex Luna C18(2) column (150 × 3 mm, particle 3 µm) at a flow rate of 300 µL/min at 35 °C (column thermostat SICO-100). Mobile phases consisted of A (0.1% formic acid in water) and B (0.1% formic acid in methanol). The linear gradient mobile phase protocol was as follows: 0–0.2 min, 10% B; 0.2–6 min, 10–95% B; 6–10 min, 95% B; 10–11 min, 10% B; 11–15 min, 10% B. The MS data were processed and analyzed using Xcalibur 3.0.63. Molecular ion adducts such as [M + H]^+^, [M + Na]^+^, [2M + H]^+^, [2M + Na]^+^, [M − H]^−^, and [2M − H]^−^ were identified, and potential compounds were searched by using the Scifinder^n^ database [34].

### 4.5. Oxygen Radical Absorption Capacity (ORAC)

The antioxidant activity was tested using the standard ORAC assay [65]. The crude extracts were analyzed using binary dilution in the concentration range of 0.1–2500 mg/L in 3 replicates. The ability of samples to absorb the generated radicals was monitored by measuring the fluorescence (excitation/emission 485/535 nm), recorded at 5 min intervals for 2 h using a SpectraMax i3x microplate reader (Molecular Devices, San Jose, CA, USA). The EC_50_ values were determined according to the concentration range using AAT Bioquest EC_50_ calculator [66].

### 4.6. Cytotoxicity Assay

The cytotoxicity assay was performed according to the standard protocol [67]. We used the following human cell lines: HepG2 (hepatocellular carcinoma), HeLa (cervical adenocarcinoma), HaCat (keratinocytes), and HEK293T (embryonal kidney cells) purchased from American Type Culture Collection ATCC (VA, USA). The cells were counted using an Auto T4 Cellometer (Nexcelom Bioscience, Lawrence, MA, USA). We seeded 1 × 10^5^ cells/mL into a 96-well plate containing 100 μL of the medium. The medium was changed after 24 h, and the crude extracts were added in the concentration range of 125–500 mg/L in 4 replicates. After 72 h of incubation, a standard resazurin assay was performed. The fluorescence was measured using a SpectraMax i3x microplate reader (Molecular Devices, San Jose, CA, USA) at a wavelength of 560/590 nm excitation/emission.

### 4.7. Modulation of Acetylcholinesterase Activity

The Ellman colorimetric method [68] was used to measure the activity of acetylcholinesterase (AChE). The crude extracts were analyzed using binary dilution in the concentration range of 62.5–0.125 mg/L. The AChE was added to the reaction mixture and the plate was incubated (37 °C, 5% CO_2_) for 15 min. After the incubation with AChE (2.5 U/well), the reaction was started by the addition of 5,5′-dithiobis-2-nitrobenzoic acid (DTNB, 0.02 g/L) and acetylcholine chloride (0.01 g/L). The absorbance was recorded using a SpectraMax i3x microplate reader (Molecular Devices, San Jose, CA, USA) at a wavelength of 412 nm at 1 min intervals for 10 min.

### 4.8. Anti-Inflammatory Activity

Nitrate oxide was quantified in the supernatant of LPS-stimulated (100 ng/L) macrophages (RAW 264.7) cultivated in MEM medium after 24 h. The supernatant was mixed with Griess reagent (0.04 g/L) at a 1:1 ratio, and the absorbance (540 nm) was measured after 15 min of incubation at room temperature. The viability of cells was verified by the resazurin assay described above.

Both IL-6 and TNF-α were quantified by ELISA. Clear Flat-Bottom Immuno Nonsteril 96-well plates (Thermo Fisher Scientific) were coated with 100 µL/well of capture antibody (TNF-α Monoclonal Antibody (TN3-19.12) or IL-6 Monoclonal Antibody (MP5-20F3), Thermo Fisher Scientific) in phosphate-buffered saline (PBS, 2 µg/mL). After overnight incubation at 4 °C, the plates were washed 3 times using wash buffer (PBS supplemented with 0.05% Tween20). The plates were then blocked with blocking buffer (20% FBS, 0.1% Tween20, PBS). The supernatant of LPS-induced (100 ng/mL) RAW 264.7 cells were collected by centrifugation after 6 h of incubation, and 10 µL was mixed with 90 µL of blocking buffer and applied to primary antibody-coated plates. After overnight incubation at 4 °C, the plates were washed and secondary antibody was added (TNF-α Polyclonal Antibody, Biotin, eBioscience or IL-6 Monoclonal Antibody (MP5-32C11), Biotin, eBioscience, 1 µg/mL, Thermo Fisher Scientific). After 1 h of incubation at room temperature, the washed plates were loaded with streptavidin–horseradish peroxidase (HRP, 1.25 µg/mL, Thermo Fisher Scientific). After 30 min of incubation at room temperature, the washed plate was loaded with tetramethyl benzidine (TMB) solution (Sigma-Aldrich). The reaction was stopped after 15 min with 1M H_3_PO_4_, and the absorbance (450 nm) was recorded, subtracting the absorbance at 570 nm.

### 4.9. Statistical Analysis and Correlation

The experiments were performed with the appropriate number (*n*) of repetitions, which are presented in each figure. The EC_50_/IC_50_ values were obtained using the software GraphPad Prism 7 (GraphPad Software, San Diego, CA, USA). The data are presented as the averages of the repetitions with the standard error of the mean (SEM). One-way analysis of variance (ANOVA) was used, followed by Tukey’s post hoc test (*p* < 0.05 or *p* < 0.01) to show the differences between the groups. Denoting the statistically significant levels with different letters is a way to summarize the differences between the extracts. If the two extracts share at least one letter, it means that they are not significantly different. It also reflects the comparison of every extract to every other extract in the same assay (column in the figure). For ANOVA, the software Statistica version 13 was used (Tibco Software Inc., Tulse, OK, USA). The selectivity index (SI) was calculated using the formula SI = IC_50_ (control cells)/IC_50_ (cancer cells).

Pearson’s correlation coefficients were calculated using the automatic function “CORREL” in Microsoft Office Excel. The analytical standards of identified components were not available, and thus we correlated either the areas of the peaks or the sum of peak areas belonging to the respective chemical class (alkaloids, fatty acids, flavonoids, (neo)lignans, phenols, quinones, selaginellins, steroids, terpenoids), which served as matrix I. The results of the biological activity of 16 *Selaginella* extracts obtained from all assays investigated (ORAC assay, selectivity index, cytotoxicity, inhibition of AChE, inhibition of NO, TNF-α and IL-6 production) were utilized as matrix II. The significance of the correlation coefficient was evaluated by using a comparison of coefficients and the critical values (α = 0.05 or lower), which were determined using the degrees of freedom (df = *n*−2).

## 5. Conclusions

*Selaginella* is well known for its unique natural products and its wide range of biological effects, which is why numerous *Selaginella* species are used in traditional medicines for the treatment of various diseases. Here, we compared the antioxidant, anti-inflammatory, and acetylcholinesterase inhibition activity as well as the cytotoxicity against cancer cell lines of eight *Selaginella* species, which highlighted *S. apoda* species among all the tested activities. The correlations of biological activity and composition of extracts verified the activity of several known biologically active compounds and for the first time highlighted several compounds for their previously unknown activities. This paper is one of the first reporting the selective cytotoxicity of *Selaginella* extracts against cancerous cells. However, significantly deeper studies are needed to confirm the compounds responsible for the activities, to determine the selectivity against various tumors derived from different tissues, and also to determine the mechanism of action.

## Figures and Tables

**Table 1:** Number of metabolites detected in methanolic extracts of *Selaginella* species.

Metabolites	*S. apoda*	*S. biformis*	*S. cupressina*	*S. delicatula*	*S. erythropus*	*S. myosuroides*	*S. uncinata*	*S. ramosii*
Alkaloids	2	2	1	4	1	0	2	2
Fatty acids	3	1	2	3	3	3	2	2
Flavonoids	36	37	27	33	35	25	32	29
Lignans/neolignans	20	15	7	9	16	10	15	6
Phenols	19	15	11	14	11	11	11	12
Quinones	1	1	1	1	1	1	0	1
Selaginellins	13	12	7	8	7	4	11	8
Steroids	4	3	2	3	1	0	3	2
Terpenoids	1	1	2	2	2	2	3	2
Unidentified	3	2	1	1	2	2	2	2
Total	102	89	61	78	79	58	81	66

**Table 2:** Numbers of metabolites detected in dichloromethane extracts of *Selaginella* species.

Metabolites	*S. apoda*	*S. biformis*	*S. cupressina*	*S. delicatula*	*S. erythropus*	*S. myosuroides*	*S. uncinata*	*S. ramosii*
Fatty acids	2	1	1	1	2	3	2	2
Flavonoids	3	5	4	5	7	7	7	7
Lignans/neolignans	1	1	5	5	6	7	5	5
Phenols	3	4	3	4	6	7	4	7
Quinones	1	1	1	1	1	1	1	1
Selaginellins	1	3	3	3	5	4	4	5
Steroids	2	2	0	0	4	3	2	5
Terpenoids	0	0	0	0	0	0	0	1
Unidentified	2	6	2	1	10	14	12	13
Total	15	23	19	20	41	46	37	46

**Table 3:** Pearson’s correlation coefficient demonstrating relationship between chemical composition and cytotoxicity against cancer cell lines of crude extracts.

	Value	*n*	df	Crit. Value	α
HeLa					
5,5′′,7,7′′,4′,4′′′-Hexahydroxy-(2′,6′′)-biflavone or 5,5′′,7,7′′,4′,4′′′-Hexahydroxy-(2′,8′′)-biflavone	0.759	7	5	0.754	0.05
Selaginpulvilin F or selaginpulvilin K or selaginellin Q	0.759	7	5	0.754	0.05
Selaginellin O or selaginpulvilin B	0.999	3	1	0.997	0.05
Selaginpulvilin E or selaginpulvilin L or selaginellin P	0.999	3	1	0.997	0.05
Selaginpulvilin I or selaginellin W	0.997	3	1	0.997	0.05
**SI (HaCat/HeLa)**					
Seladoeflavone E	0.982	4	2	0.980	0.02
Sinensioside A	1.000	3	1	1.000	0.02
**SI (HEK 293T/HepG2)**					
Pinocembrin-7-O-β-d-glucopyranoside	0.733	8	6	0.707	0.05
Compound **1** *	0.733	8	6	0.707	0.05
Methyl cinnamate	0.733	8	6	0.707	0.05
Compound **2** *	0.895	6	4	0.882	0.02
Oleic acid	0.892	5	3	0.878	0.05
Chalcone	0.864	8	6	0.834	0.01
5-Carbomethoxymethyl-4′,7-dihydroxyflavone	0.814	6	4	0.811	0.05
Phellodensin F	0.774	8	6	0.707	0.05
Compound **3** *	0.789	8	6	0.789	0.02
Viburnolide C	0.774	8	6	0.707	0.05
1-Methoxy-3-methylanthraquinone	0.789	8	6	0.789	0.02
Compound **4** *	0.998	3	1	0.997	0.02
**HEK 293T**					
Compound **5** *	0.999	3	1	0.997	0.05

* Compound **1**: 6-(5-acetyl-2-methoxyphenyl)-5,7-dihydroxy-2-(4-hydroxyphenyl)-4*H*-chromen-4-one or (7S,8R)-4,9-dihydroxy-3,3′,5-trimethoxy-4′,7-epoxy-8,5′-neolignan-9′-oic acid methyl ester (= (2S,3R)-2,3-dihydro-2-(4-hydroxy-3,5-dimethoxyphenyl)-3-(hydroxymethyl)-7-methoxybenzofuran-5-propanoic acid methyl ester. Compound **2**: 6-formyl-5-isopropyl-3-hydroxymethyl-7-methyl-1*H*-indene. Compound **3**: (3R)-5,6,7-trihydroxy-3-isopropyl-3-methylisochroman-1-one. Compound 4: 3β,4β,23-trihydroxy-24,30-dinorolean-12,20(29)-dien-28-oic acid. Compound **5**: (3R,4S)-dihydro-4-hydroxy-3,4-bis[(4-hydroxy-3-methoxyphenyl)methyl]-2(3*H*)-furanone or 1-[(2R,3S)-2,3-dihydro-2-(4-hydroxy-3,5-dimethoxyphenyl)-3-(hydroxymethyl)-7-methoxy-5-benzofuranyl]ethanone. Value is the value of the Pearson’s correlation coefficient, *n* is the number of repetitions, df is the degree of freedom calculated as *n*−2, Crit. Value is the value stated in the table of the Pearson’s correlation coefficient at selected significance level (α).

**Table 4:** Pearson’s correlation coefficient demonstrating relationship between chemical composition and anti-inflammatory activity of crude extracts.

	Value	*n*	df	Crit. Value	α
NO production					
Lignans or neolignans [39]	0.856	8	6	0.834	0.01
Seladoeflavone E	0.920	5	3	0.878	0.05
Selamoellenin A	0.920	5	3	0.878	0.05
**TNF-α production**					
Robustaflavone 4′-methyl ether or podocarpusflavone or neocryptomerin or sequoiaflavoneor isocryptomerin or sotetsuflavone	0.989	5	3	0.934	0.02
Selagin	0.908	6	4	0.811	0.05
3-Hydroxy-4-carboxy-2-methoxyphenyl ester benzenepropanoic acid	0.908	6	4	0.811	0.05
Pulvinataphendiol	0.908	6	4	0.811	0.05
**IL-6 production**					
Selagin	0.943	6	4	0.917	0.01
3-Hydroxy-4-carboxy-2-methoxyphenyl ester benzenepropanoic acid	0.943	6	4	0.917	0.01
Pulvinataphendiol	0.943	6	4	0.917	0.01
Daucosterol	0.813	6	4	0.811	0.05

The slashes between the compounds in a row reflects the fact that these are indistinguishable by the mass spectrometry. Value is the value of the Pearson’s correlation coefficient, *n* is the number of repetitions, df is the degree of freedom calculated as *n*−2, Crit. Value is the value stated in the table of the Pearson’s correlation coefficient at selected significance level (α).

## Data Availability

The data presented in this study are available in this article or associated supplementary material.

## Supplementary Materials

The following are available online at https://www.mdpi.com/1424-8247/14/1/16/s1: Figure S1: Cytotoxicity against cancer cell lines and antioxidant, anti-inflammatory, and anti-acetylcholinesterase activity of appropriate standards: (a) doxorubicin, (b) quercetin, (c) indomethacin, and (d) eserin. Table S1: Overview of phytochemicals detected and tentatively identified in *Selaginella* by ultra-high performance liquid chromatography-high-resolution mass spectrometry (UHPLC–HRMS). Figure S2: Chemical structures of pharmacologically active ingredients mentioned in Table 3 and Table 4, which are unique for *Selaginella* species.
