# Supplementary material for: Comparison of Chemical Composition and Biological Activities of Eight Selaginella Species
Source: Pharmaceuticals (Basel). 2020 Dec 26;14(1):16. doi: 10.3390/ph14010016 (PMC7823444; doi:10.3390/ph14010016)
Supplement: Supplementary file 1 [file pharmaceuticals-14-00016-s001.zip › Fig S2.docx]

Figure S2: Chemical structures of secondary metabolites identified from *Selaginella* extracts
