# Supplementary material for: Comparison of Chemical Composition and Biological Activities of Eight Selaginella Species
Source: Pharmaceuticals (Basel). 2020 Dec 26;14(1):16. doi: 10.3390/ph14010016 (PMC7823444; doi:10.3390/ph14010016)
Supplement: Supplementary file 1 [file pharmaceuticals-14-00016-s001.zip › Supp.Fig.1.docx]

(a) (b)

(c) (d)

(e)

Figure S1: Anticancer, antioxidant, anti-acetylcholinesterase and anti-inflammatory activity of appropriate standards: a) doxorubicin, selectivity index (ratio of IC_50_ values of HEK293T and HepG2 cell lines) = 7.2±0.7; b) doxorubicin, selectivity index (ratio of IC_50_ values of HDF and HeLa cell lines) = 0.1±0.01; c) quercetin, IC_50_ = 2.4±0.2 µM; d), eserine, IC_50_ = 6.3±0.3 µM; e) indomethacin, IC_50_ = 57.2±3.6 µM.
